# Metagenomic Analysis of the Ferret Fecal Viral Flora

**DOI:** 10.1371/journal.pone.0071595

**Published:** 2013-08-20

**Authors:** Saskia L. Smits, V. Stalin Raj, Minoushka D. Oduber, Claudia M. E. Schapendonk, Rogier Bodewes, Lisette Provacia, Koert J. Stittelaar, Albert D. M. E. Osterhaus, Bart L. Haagmans

**Affiliations:** 1 Department of Viroscience, Erasmus Medical Center, Rotterdam, The Netherlands; 2 Viroclinics Biosciences BV, Rotterdam, The Netherlands; Columbia University, United States of America

## Abstract

Ferrets are widely used as a small animal model for a number of viral infections, including influenza A virus and SARS coronavirus. To further analyze the microbiological status of ferrets, their fecal viral flora was studied using a metagenomics approach. Novel viruses from the families *Picorna-, Papilloma-,* and *Anelloviridae* as well as known viruses from the families *Astro-, Corona-, Parvo-*, and *Hepeviridae* were identified in different ferret cohorts. Ferret kobu- and hepatitis E virus were mainly present in human household ferrets, whereas coronaviruses were found both in household as well as farm ferrets. Our studies illuminate the viral diversity found in ferrets and provide tools to prescreen for newly identified viruses that potentially could influence disease outcome of experimental virus infections in ferrets.

## Introduction

Infectious viral diseases, both emerging and re-emerging, pose a continuous health threat and disease burden to humans. Many important human pathogens are zoonotic or originated as zoonoses before adapting to humans [1]–[4]. This is exemplified by recently emerged diseases in which mortality ranged from a few hundred people due to infection with H5N1 avian influenza A virus to millions of HIV-infected people from acquired immunodeficiency syndrome [5]–[8]. Severe acute respiratory syndrome (SARS) coronavirus and the pandemic influenza A/H1N1(2009) virus in humans were linked to transmission from animal to human hosts as well and have highlighted this problem [9]–[11]. An ongoing systematic global effort to monitor for emerging and re-emerging pathogens in animals, especially those in key reservoir species that have previously shown to represent an imminent health threat to humans, is crucial in countering the potential public health threat caused by these viruses.

Relatively few studies have been conducted on diseases of non-domestic carnivores, especially regarding diseases of small carnivores (e.g. mustelids). Ferrets (*Mustela putorius furo*) can carry bacteria and parasites such as *Campylobacter, Giardia,* and *Cryptosporidium* in their intestinal tract and potentially spread them to people [12], [13]. In addition, they can transmit influenza A virus to humans and possibly on rare occasions rabies virus as well [14]–[16]. Because of their susceptibility to several human respiratory viruses, including human and avian influenza viruses, SARS coronavirus, nipah virus, and morbilliviruses [17]–[21], ferrets have been used as small animal model for these viruses. To further characterize this important animal model and to obtain epidemiological baseline information about pathogens in ferrets, the fecal viral flora of ferrets was studied using a metagenomics approach. Both known and new viruses were identified.

## Materials and Methods

### Clinical specimens

Rectal swabs were collected from 39 ferrets (*Mustela putorius furo*) in the Netherlands and Sweden and stored at −80°C (Table 1) from 2010–2012. The first cohort consisted of rectal swabs from ferrets from a farm in The Netherlands in 2010 (*n* = 12). The second cohort consisted of rectal swabs from ferrets (Sweden) that were used in an experimental procedure that consisted of 3 weeks of immunosuppression [22] gathered in March 2012 (*n* = 12). The third cohort were rectal swabs from household ferrets (the Netherlands) with (*n* = 3) or without (*n* = 12) diarrhea gathered in 2010. This study was carried out in strict accordance with European guidelines (EU directive on animal testing 86/609/EEC) and Dutch legislation (Experiments on Animals Act, 1997). In compliance with relevant laws and institutional guidelines, no ethical approval is required for most of the samples in this study, because live animals were not primarily and purposefully sampled to obtain material for scientific purposes, but for health care purposes, such as surveillance of viruses that could cause underlying ferret disease. Some of the samples from the ferrets in this study were obtained under ethical approval by the independent animal experimentation ethical review committee of the Netherlands Vaccine Institute (permit number 20110286) in compliance with relevant laws and institutional guidelines. All efforts were made to minimize animal suffering and the owners of the ferrets gave permission for their animals to be used in this study.

**Table 1:** Summary of mammalian viruses found in ferret fecal material.

Ferret	Housing^c^	Total no. reads^a^	Eukaryotic viruses (No. of reads)	Virus in-depth^d^	Taqman^d^
1	Farm NL	3981	Picobirnavirus (2)		FRCoV
2	Farm NL	10149	Papillomavirus (3)		FRCoV
3	Farm NL	5186	Coronavirus (17)		FRCoV
			Picobirnavirus (10)		
			Kobuvirus (75)		
4	Farm NL	8919	Torque teno virus (3)		
5	Farm NL	71706			
6	Farm NL	7710			
7	Farm NL	7344			
8	Farm NL	7686	Aleutian mink disease virus (2)		FRCoV
9	Farm NL	9576			
10	Farm NL	7702	Aleutian mink disease virus (2)		FRCoV
11	Farm NL	1149			
12	Farm NL	4351			
13	Farm SE	4947			
14	Farm SE	6560			
15	Farm SE	4632	Coronavirus (116)		FRCoV
16	Farm SE	5985			
17	Farm SE	4766	Papillomavirus (92)	MpPV1	MpPV1
18	Farm SE	4728			
19	Farm SE	3641	Coronavirus (19)		FRCoV
			Kobuvirus (90)		MpKoV
20	Farm SE	5627	Kobuvirus (11)		
21	Farm SE	6091	Coronavirus (43)		FRCoV
			Torque teno virus (48)	MpfTTV1	
22	Farm SE	3422			
23	Farm SE	706			
24	Farm SE	3099	Coronavirus (22)		FRCoV
25	Household	6249			FRCoV
26	Household	7745	Astrovirus (636)		
			Coronavirus (13)		FRCoV
			Picobirnavirus (3)		
27	Household	10002			
28	Household	13634			
29	Household	4298			
30	Household	4886	Picobirnavirus (9)		
			Polyomavirus (3)		
31	Household	8959			
32	Household	8369	Kobuvirus (4280)	MpKoV32	MpKoV
			Polyomavirus (2)		
33	Household	8459	Herpesvirus (2)		
			Kobuvirus (2039)		MpKoV
			Hepevirus (562)		FRHEV
			Coronavirus (35)		FRCoV
34	Household	7116			FRCoV
35	Household	1167	Hepevirus (898)		FRHEV
36	Household	9539	Kobuvirus (50)		MpKoV
			Parechovirus (71)	MpPeV1	MpPeV1
37	Household^b^	5069	Astrovirus (1464)		
			Hepevirus (516)		FRHEV
38	Household^b^	13387	Coronavirus (19)	MpKoV38	FRCoV
			Kobuvirus (6160)		MpKoV
39	Household^b^	9117	Kobuvirus (3582)	MpKoV39	MpKoV

a Total no. of trimmed reads that were analyzed.

b These animals had diarrhea.

c NL, Netherlands; SE, Sweden.

d FRCoV, ferret coronavirus; MpPV1, ferret papillomavirus; MpKoV, ferret kobuvirus; FRHEV, ferret hepevirus; MpPeV, ferret parechovirus; MpfTTV, ferret anellovirus.

### Sequence-independent RNA and DNA virus screening of ferret rectal swabs

Large scale molecular virus screening, based on host nucleic acid depletion, viral nucleic acid isolation, sequence-independent amplification and next generation sequencing with a 454 GS Junior Instrument (Roche) was performed as described previously and by the manufacturer [23]–[26] on 39 rectal swabs from ferrets. More than 253,000 trimmed reads were assembled using de novo assembly in CLC Genomics Workbench 4.5.1 (CLC Bio; [27]) and analyzed according to nucleotide (contigs and singletons) and translated nucleotide BLAST searches (contigs) [28]. Sequences were classified into eukaryotic viruses, phages, bacteria, and eukaryotes based on the taxonomic origin of the best-hit sequence using MEGAN 4.40 [29], [30]. E values of 0.001 and 10^−10^ were used as the cutoff value of significant virus hits for BLASTn and BLASTx, respectively.

### PCR amplification and Sanger sequencing

Sequence information obtained from the large scale molecular virus screening using next-generation sequencing was used to design specific primers for amplification of the papillomavirus genome MpPV1 using AmpliTaq Gold DNA polymerase (Roche), according to instructions of the manufacturer to confirm and/or extend 454-sequence reads. Primer sequences are available on request. Products were sequenced as described previously [26]. A diagnostic MpPV1 real time PCR was developed, using primers and probe VS736 (5′-ATGTGTGACATTGGCTTTGGA- 3′), VS738 (5′-CTGCAATATGACTGCTCTCGC- 3′), and VS737 (5′-FAM-CTAGAGCTGAGGTTCCTATAG-TAMRA-3′) that target the L1 genome region (KF006988) and TaqMan® Fast Virus 1-Step Master Mix (Applied Biosystems) according to instructions of the manufacturer. In addition, diagnostic ferret kobu- and parechovirus real time RT-PCRs were developed, using primers and probes VS730 (5′-CCTCCAGCTCCGCTGGCTCAA-3′), VS732 (5′-CAATCCTAGGGTGCAGTCCTT- 3′), and VS731 (5′-FAM- CCACTCTCTCTGTGGCACTTT-TAMRA- 3′) that target the 3D polymerase/3′UTR genome region of ferret kobuvirus (KF006985) and primers and probe VS733 (5′-CTGCTCCTCAATTAACAGGCT-3′), VS735 (5′-CACACTCCTGCATCCATTAGT- 3′), and VS734 (5′-FAM-TGAGATCATTGCATGACAATGT-TAMRA- 3′) that target the 3D genome region of ferret parechovirus (KF006989) with TaqMan® Fast Virus 1-Step Master Mix (Applied Biosystems) according to instructions of the manufacturer. Similarly, a diagnostic ferret hepatitis E virus real time RT-PCR was used, using primers and probe VS739 (5′- AAGATGCGTTTTGTTCTCTTGCT-3′), VS740 (5′- CCGGACGCCCTCCTGYA- 3′), and VS741 (5′-FAM- TGCTCGCCGCCCCAATGTACC-TAMRA- 3′) that target the ORF2/3 genome region of ferret hepatitis E virus (JN998606). A diagnostic ferret coronavirus real time RT-PCR was used with primers and probe as described previously (22).

### Phylogenetic analysis

Multiple alignments were created using ClustalX (2.0.10) [31]. Phylogenetic analyses were carried out with Molecular Evolutionary Genetics Analysis (MEGA), version 5 [32]. Picornavirus cleavage sites were predicted by NetPicoRNA 1.0 server (http://www.cbs.dtu.dk/services/NetPicoRNA/) or predicted based on alignments with other picornaviruses. The similarity between different picornaviruses was measured using Simplot version 3.5.1 with the Kimura-2 parameter, a transition/transversion (Ts/Tv) ratio of 3.0 and window and step sizes of 600 and 20 nucleotides, respectively [33].

### Genbank accession numbers

The genomes of identified viruses described in detail here were deposited in GenBank under the following accession numbers: ferret kobuviruses MpKoV38, KF006985; MpKoV32, KF006987; MpKoV39, KF006986; ferret parechovirus (MpPeV1), KF006989; ferret papillomavirus (MpPV1), KF006988; ferret anellovirus (MpfTTV1), KF006990.

## Results

Large scale molecular virus screening was performed on 39 rectal swabs from ferrets (*Mustela putorius furo)*. More than 253,000 trimmed sequence reads were generated and the sequences from each animal were assembled de novo. Sequences were classified into eukaryotic viruses, phages, bacteria, and eukaryotes based on the taxonomic origin of the best-hit sequence. Many of the identified sequences were of eukaryotic or bacterial origin, and a large proportion of the total reads did not have any significant hits for nucleotide or amino acid sequences in GenBank in agreement with viral metagenomic studies of feces from bats, turkeys, rodents, pine marten, and humans [25], [34]–[36]. Fecal samples of ferrets revealed a large degree of microbial diversity.

### Virome overview

**Figure 1:**
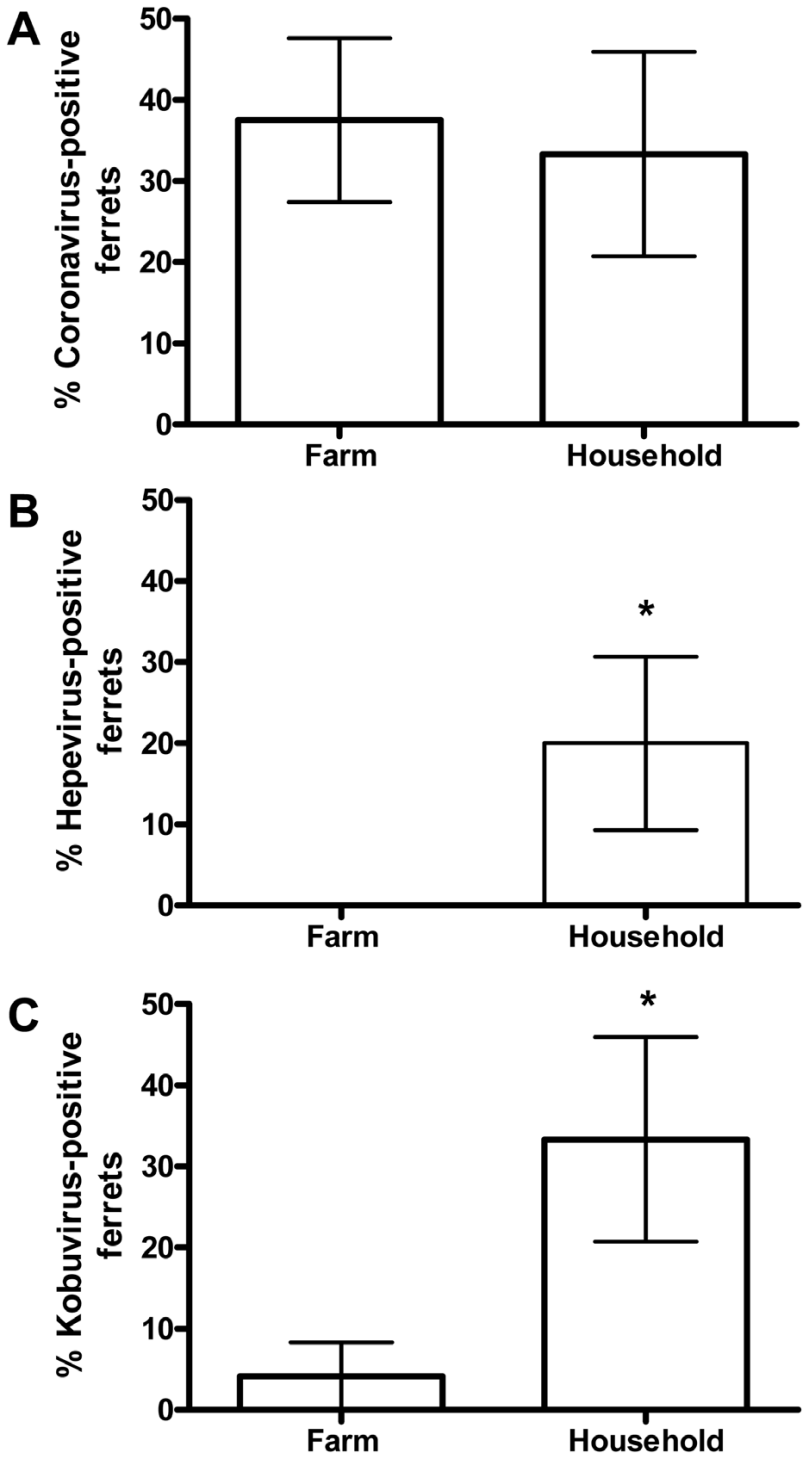
Prevalence of ferret viruses. Percentage coronavirus (A), hepatitis E virus (B) and kobuvirus (**C**) positive farm versus household ferrets by real time PCR assay. Significant differences (unpaired t-test P<0.05) are indicated by an asterisk.

Almost all samples showed evidence for the presence of bacteriophages from the order *Caudovirales* and/or family *Microviridae*. In seven rectal swabs, evidence for quite a number of different avian viruses, among which chicken anemia virus, chicken astrovirus, chicken parvovirus, turkey hepatitis virus, fowl adenovirus, and turkey parvovirus. Six of these animals were bred on a farm and one animal was a household pet. These avian viruses may originate from the ferrets'diet (they were fed chicken) and do not represent genuine enteric infections in the ferrets. Eukaryotic viruses with relatively high homology (85–100% on the amino acid level) to ferret coronavirus ([37]; family *Coronaviridae*), ferret hepatitis E virus ([38]; family *Hepeviridae*), and Aleutian mink disease virus (family *Parvoviridae*), were detected (Table 1). The species demarcation criteria are not strongly defined for *Corona*- and *Hepeviridae*, but based on host range and genetic similarity they should qualify as the same species as ferret coronavirus and ferret hepatitis E virus. The presence of corona- and hepeviruses was confirmed by real time PCR assays (Table 1). Of note, ferret hepatitis E virus sequences were only detected in household ferrets, whereas coronaviruses were found both in household as well as farm ferrets, (Fig. 1; Table 1). A near complete and a partial genome of an astrovirus (family *Astroviridae*) were obtained from ferrets 37 and 26 respectively with ∼95% identity on the nucleotide level to murine astrovirus STL1 that was recently described (Table 1) [39], [40]. In addition, a number of ferret viruses with homology to torque teno virus (family *Anelloviridae*), picobirnavirus (family *Picobirnaviridae*), kobu- and parechovirus (family *Picornaviridae*), and papillomavirus (family Papillomaviridae) were identified (Table 1). We further characterized some of these novel mammalian viral sequences by full or near full genome sequencing and compared them to their closest relatives by phylogenetic analyses.

### Ferret kobuvirus

Kobuvirus is a viral genus belonging to the family *Picornaviridae*. The genus is composed of three species, called Aichivirus A, B, and C. Aichiviruses A, B, and C have been found in humans [41], cows [42], and pigs [43], respectively. More recently, kobuviruses from Canyon mouse (*Peromyscus crinitus*) [44], dogs (*Canis lupus familiaris*) [45], [46] and sheep [47] have been identified. This virus may be associated with diarrhea [48]. Kobuviruses are small, nonenveloped viruses with a single stranded positive sense RNA genome of ∼8 kb. The genome encodes a single polyprotein that is cleaved by virus-encoded proteases into the structural and nonstructural proteins [48].

**Figure 2:**
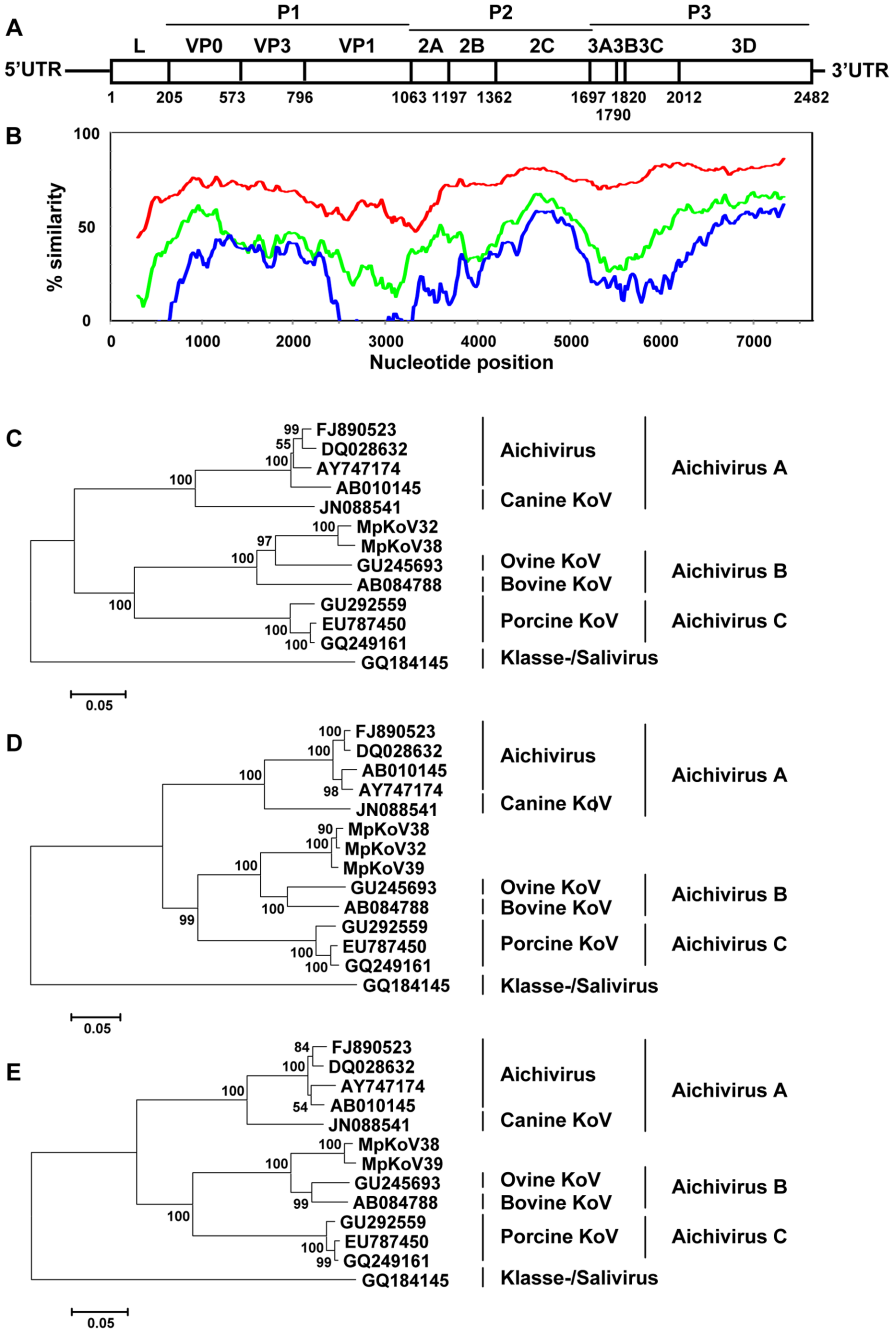
Genome organization of ferret kobuvirus and amino acid sequence divergence from other aichiviruses. (A) Predicted genome organization of ferret kobuvirus showing amino acid positions of predicted cleavage sites in the polyprotein (numbering based on the ferret kobuvirus polyprotein sequence). Sites were predicted by NetPicoRNA analysis and by alignment with known cleavage sites in aichiviruses. (B) Mean similarity of bovine kobuvirus (AB084788) to ferret kobuvirus (red), porcine kobuvirus (GU292559, green), and human aichivirus (FJ890523; blue) polyprotein-coding nucleotide sequences scaled to the genome diagram in A. (C–E) Phylogenetic trees of the amino acid sequences of ferret kobuvirus (MpKoV32, 38, and 39) with other aichiviruses in the P1 (C), P2 (D), and P3 (E) gene regions were generated using MEGA5, with the neighbor-joining method with p-distance and 1,000 bootstrap replicates. Significant bootstrap values are shown.

A near-complete kobuvirus genome containing the complete coding region was obtained by 454-sequencing from a rectal swab from ferret 38. In addition, two partial polyprotein sequences were obtained from rectal swabs of household ferrets 32 and 39. The ferret kobuvirus (MpKoV) from ferret 38 encodes a 2,482-amino-acid polyprotein flanked on each side by untranslated regions (UTRs) (Fig. 2A). Polyprotein cleavage sites were predicted by NetPicoRNA analysis and based on kobuvirus alignments (Fig. 2A). NetPicoRNA predictions were strong for the L/VP0, VP1/2A, 2A/2B, 2B/2C, and 2C/3A, all containing Q/G residues on either side of the predicted site. A Q/S cleavage site between 3C and 3D equivalent to that of aichiviruses was predicted at position 2012–2013 of MpKoV38. Q/H and Q/A cleavage sites in the P1 region were predicted by comparison with aichivirus sites. The 3A/3B and 3B/3C sites were tentatively placed at position 1790 (DQAQ/AAYT) and at position 1820 (VVRQ/SGPS) based on alignments with other kobuviruses.

The genetic relationship of MpKoV38 with other known kobuviruses was assessed by determining the pairwise distance to the corresponding genome regions of other picornaviruses (Table 2; Fig. 2B–E). MpKoV38 showed the greatest sequence identity with bovine and ovine kobuvirus throughout the genome (Table 2; Fig. 2B–E). According to the 9th ICTV Report [49], members of kobuvirus species share greater than 70% amino acid identity in the polyprotein, greater than 70% amino acid identity in P1 genome region, greater than 80% amino acid identity in 2C and 3CD genome regions, and share a common genome organization, suggesting that ferret kobuvirus belongs to aichivirus species B. The diversity of ferret kobuvirus from bovine and ovine kobuviruses, however, was consistently greater than that observed within different isolates of human aichiviruses or different ferret kobuvirus isolates, confirming its identity as a separate type (Fig. 2C–E). No evidence of genetic recombination among ferret KoV or other kobuviruses was observed (Table 2, Fig. 2B–E).

**Table 2:** Pairwise amino acid identity in the P1, P2, and P3 regions between ferret kobuvirus 38 (MpKoV38; KF006985) and bovine kobuvirus (BKoV; AB084788), human aichivirus (AIV; FJ890523), porcine kobuvirus (PKoV; GU292559), and klassevirus (Klasse; GQ184145).

Region and virus	BKoV	AIV	PKoV	Klasse
P1				
MpKoV38	80.8	52.1	63.2	41.7
BKoV		52.2	63.1	41.7
AIV			56.2	44.2
PKoV				41.9
P2				
MpKoV38	83.3	62.7	70.4	36.0
BKoV		62.4	72.0	35.5
AIV			63.7	34.2
PKoV				35.4
P3				
MpKoV38	89.5	63.8	72.3	42.8
BKoV		64.5	74.0	42.5
AIV			66.0	45.0
PKoV				43.3

To obtain insight in the prevalence of ferret kobuvirus in ferrets, a ferret kobuvirus-specific real time PCR targeting the 3D/3′NTR region of the kobuvirus genome was performed on the total set of 39 rectal samples. Besides samples 32, 38, and 39, in which ferret kobuvirus was identified, three other rectal samples were positive as well, resulting in an overall prevalence of ∼15% (Table 1; Fig. 1). As was observed for ferret hepatitis E virus, ferret kobuvirus is significantly more often detected in household ferrets than in farm ferrets using a real time PCR assay (Fig. 1).

### Ferret parechovirus

Parechovirus is a viral genus in the family *Picornaviridae*. The genus is composed of two species, human parechovirus and Ljungan virus from bank voles. Parechoviruses are small, non-enveloped viruses with a single stranded positive sense RNA genome of ∼7.5 kb. The genome encodes a single polyprotein that is cleaved by virus-encoded proteases into the structural and nonstructural proteins. Human parechoviruses are widely spread infectious pathogens that are associated with mild gastroenteritis, respiratory disease, flaccid paralysis, encephalitis, and myocarditis in young children [50]–[53].

**Figure 3:**
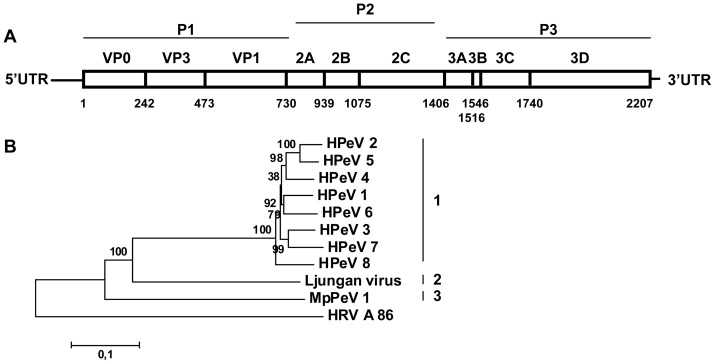
Genome organization and phylogenetic analysis of ferret parechovirus. (A) Predicted genome organization of ferret parechovirus showing amino acid positions of predicted cleavage sites in the polyprotein (numbering based on the ferret parechovirus polyprotein sequence). Sites were predicted by NetPicoRNA analysis and by alignment with other parechoviruses. (B) A phylogenetic tree of the polyprotein sequence of ferret parechovirus (MpPeV1) and representative human (HPeV1-8) and bank vole parechoviruses (Ljungan virus) was generated using MEGA5, with the neighbor-joining method with p-distance and 1,000 bootstrap replicates and human rhinovirus A 86 as an outgroup (HRV-A 86). Significant bootstrap values are shown. Genbank accession numbers are shown in Table S1.

A near-complete parechovirus genome containing the complete coding region was obtained by 454-sequencing from a rectal swab from household ferret 36 without diarrhea. The ferret parechovirus from ferret 36 (MpPeV1) encodes a 2,207-amino-acid polyprotein flanked on each side by untranslated regions (UTRs) (Fig. 3A). Cleavage sites were predicted by NetPicoRNA analysis and alignments with other parechoviruses (Fig. 3A). The pairwise identity of the MpPeV1 polyprotein with the corresponding genome regions of other parechoviruses was assessed (Table 3; Fig. 3B). The *Picornavirus* Study Group of ICTV determined that members of a species of the parechovirus genus share greater than 70% amino acid identity in the polyprotein, and share a natural host range and genome organization. Members of a picornavirus genus should normally share phylogenetically related P1, P2 and P3 genome regions, each sharing >40%, >40% and >50% amino acid identity, respectively [49]. MpPeV1 has a typical parechovirus genome organization (Fig. 2A), but shares less than 43% identity on the amino acid level in the entire polyprotein compared to human parechoviruses and Ljungan virus (Table 3) and has been identified in a host different from bank vole and human and thus MpPeV1 constitutes a new species in the genus parechovirus, and may even represent the first species in a new genus in the *Picornaviridae* family.

**Table 3:** Pairwise amino acid identity in the polyprotein P1, P2, and P3 regions between different parechovirus species.

Region and virus	HPeV1	HPeV2	Ljungan
P1			
MpPeV1	40.1	40.3	43.1
HPeV1		79.3	50.5
HPeV2			50.4
P2			
MpPeV1	40.6	40.1	46.2
HPeV1		94.9	47.7
HPeV2			48.8
P3			
MpPeV1	37.5	37.5	40.0
HPeV1		93.6	46.3
HPeV2			46.6

HPeV1 (JX575746), HPeV2 (AF055846), Ljungan (NC_003976), MpPeV1 (KF006989).

To obtain insight in the prevalence of MpPeV1 in ferrets, a MpPeV1-specific real time PCR targeting the 3D region of the parechovirus genome was performed on the total set of rectal samples from 39 ferrets. Besides the sample in which MpPeV1 was identified, no samples were positive (Table 1).

### Ferret papillomavirus

Papillomaviruses (PVs) are a highly diverse family of viruses with double stranded circular DNA genomes of ∼8 kb in size. They infect a wide variety of mammals, as well as birds and reptiles, and are highly species specific. Some papillomaviruses cause benign or malignant epithelial tumors of the skin and mucous membranes in their natural hosts [54].

**Figure 4:**
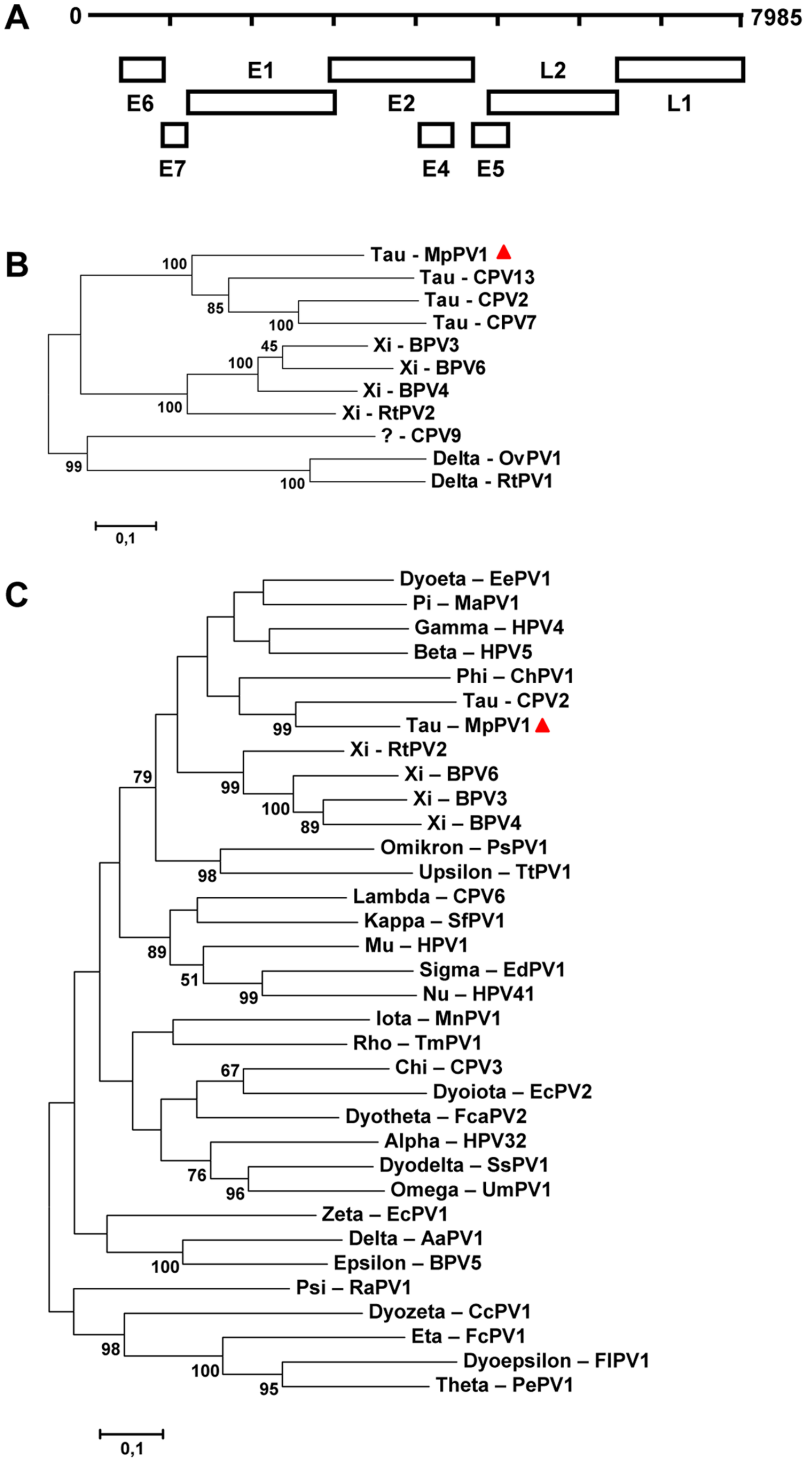
Genome organization of ferret papillomavirus and nucleotide sequence divergence from other papillomaviruses. (A) Predicted genome organization of ferret papillomavirus with early (E) and late (L) genes indicated. (B) A phylogenetic tree of the complete ferret papillomavirus (MpPV1) genome and representative human and animal papillomaviruses was generated using MEGA5, with the maximum-likelihood method with Kimura-2 parameter and 1,000 bootstrap replicates. Significant bootstrap values are shown. (C) A phylogenetic tree of the L1 genome region of ferret papillomavirus (MpPV1) and representative human and animal papillomaviruses was generated using MEGA5, with the maximum-likelihood method with Kimura-2 parameter and 1,000 bootstrap replicates. Significant bootstrap values are shown. Genbank accession numbers are shown in Table S1.

A full-length papillomavirus genome sequence was amplified and sequenced from a rectal swab of ferret 17, to complete and/or confirm the findings by next-generation sequencing. The circular genome organization of the ferret papillomavirus (MpPV1) was typical for papillomaviruses, consisting of a long control region (LCR), and early (E6, E7, E1, E2, E4) and late regions (L1, L2) (Fig. 4A). Whole genome sequence alignments revealed that the most closely related papillomavirus to MpPV1 was canine papillomavirus 2 (60% identity) from the genus *Taupapillomavirus* (Fig. 4B). Papillomaviruses are classified based on nucleotide sequence identity in the L1 ORF and their biological and pathological properties [54], [55]. A papillomavirus strain is a new type if the complete genome has been sequenced and the L1 ORF shares less than 90% homology with the closest known papillomavirus type [54], [55]. L1 is the most conserved region among papillomaviruses and according to the current genus classification, most papillomavirus types within a genus share more than 60% nucleotide identity in L1. Based on these criteria, MpPV1 could be designated as a new papillomavirus type in the genus *Taupapillomavirus* (Fig. 4C), as MpPV1 L1 shares 68.2% nucleotide identity to canine papillomavirus 2.

To obtain insight in the prevalence of MpPV1 in ferrets, a MpPV1-specific real time PCR targeting the L1 region of the papillomavirus genome was performed on the total set of 39 rectal samples. Besides the sample in which MpPV1 was identified, no samples were positive for MpPV1 (Table 1).

### Ferret anellovirus

**Figure 5:**
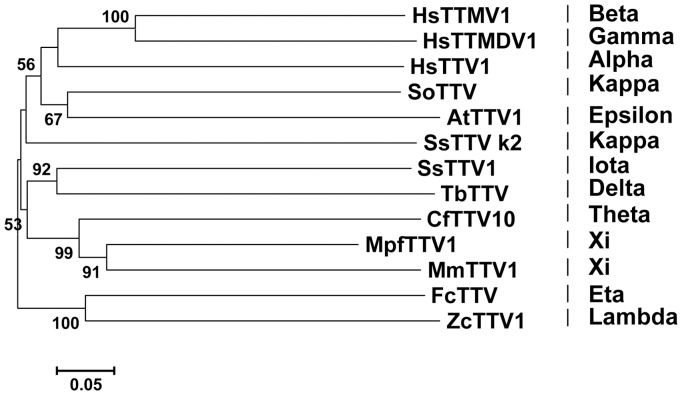
Phylogenetic analysis of ferret anellovirus. A phylogenetic tree of the partial ORF1 nucleotide sequence of ferret torque teno virus (MpfTTV1) and the corresponding region of representative human and animal anelloviruses was generated using MEGA5, with the neighbor-joining method with p-distance and 1,000 bootstrap replicates. Significant bootstrap values are shown. Genbank accession numbers are shown in Table S1.

The partial anellovirus genome sequence from sample 21 was designated MpfTTV1 for *Mustela putorius furo* torque teno virus 1. This sequence encodes for a partial open reading frame 1 (ORF1) as determined by alignment with ORF1 amino acid sequences from representative torque teno viruses. A taxonomic proposal submitted to the International Committee on the Taxonomy of Viruses (ICTV) by the *Anelloviridae-Circoviridae* Study Group proposes that genera in the family *Anelloviridae* are defined as having >56% divergence in the nucleotide sequence of ORF1 [56]. Divergence analysis using p-distance calculated with MEGA5 [32] demonstrated that the partial ORF1 nucleotide sequence of anellovirus MpfTTV1 was in general >56% divergent on the nucleotide level from anelloviruses identified in wildlife, with the exception of MmTTV1 (∼50%) (data not shown). Neighbor-joining phylogenetic trees were generated using the partial ORF1 nucleotide alignments, which underlined the nucleotide divergence analysis (Fig. 5). Our data therefore suggest that the torque teno virus species identified in ferrets belongs to the genus *Xitorquevirus*.

## Discussion

Previously, virus discovery in animals has focused on pathogenic infections or on animals that on the basis of relevance to (re-)emerging viruses are thought to represent a key risk host for emerging virus-associated disease in humans [57]. With the recent advances in the metagenomics field, a substantial increase in studies looking at virus epidemiological baseline levels in different (wildlife) animals has been observed [25], [36], [46], [57]–[68]. Even though ferrets are a very important animal model for a number of human viral infections, not much is known about viruses that naturally occur in ferrets [17]–[21]. This study describes the viral communities in fecal material of ferrets (*Mustela putorius furo*).

Sequences closely related to known viral sequences were identified, with homology to ferret coronavirus, ferret hepatitis E virus, Aleutian mink disease virus, different avian viruses and murine astrovirus STL1 [37], [38], [40]. The avian viruses may be a reflection of the diet of these ferrets, which were fed chicken. It is of note that both the viral screening with random amplification and next-generation sequencing and a specific ferret hepatitis E virus taqman assay indicated that household ferrets that are kept as pets are significantly more likely to excrete ferret hepatitis E virus in their fecal material than farm animals in this study. Such a correlation was not observed for ferret coronavirus excretion for which prevalence in both cohorts seemed similar, but not as high as previously reported [37]. Sporadic cases of human hepatitis E virus infections seem to be increasing and several observations suggest that these cases are caused by zoonotic spread of infection from wild or domestic animals [69]–[71]. Follow-up studies into seroprevalence and/or virus prevalence in household ferrets, their owners, and possibly other pets may provide indications for cross species transmission.

We report on the first identification of a kobuvirus, parechovirus, papillomavirus and anellovirus in ferrets. The kobu- and parechovirus belong to the family *Picornaviridae* that currently comprises 17 genera and many more different virus species [49]. They infect a wide range of mammals and are well-known for their involvement in gastroenteritis [49]. Our phylogenetic analysis revealed that the ferret kobuvirus clustered with bovine and ovine kobuvirus in species Aichivirus B. The close relationship between bovine, ovine, and ferret kobuviruses may indicate past cross species transmission events and subsequent evolution in the separate host species resulting in the distinct types seen today. As was observed for ferret hepatitis E virus but not ferret coronavirus, ferret kobuvirus is significantly more often detected in household ferrets than in farm ferrets by a specific ferret kobuvirus taqman assay, although random amplification and next-generation sequencing suggested the presence of kobuvirus in two additional samples from farm ferrets, which could reflect the presence of another divergent kobuvirus species. Also here follow-up studies into seroprevalence and/or virus prevalence in household ferrets, their owners, and possibly other pets should be performed. An overall kobuvirus prevalence of ∼15% was observed in this study. Two out of 3 animals (67%) with diarrhea and 4 animals out of 36 (11%) without diarrhea were positive by taqman for ferret kobuvirus. Because of the small sample size and the fact that other causes for diarrhea were not excluded, further in depth studies are needed to show that these viruses can cause diarrhea in ferrets.

The ferret parechovirus is the third parechovirus species identified to date. It is highly divergent from human parechovirus and Ljungan virus and may even constitute a new genus in the family *Picornaviridae* based on the observed sequence diversity. Interestingly, the P3 genome region of MpPeV1, encoding the nonstructural proteins NS3A–3D, is not more conserved than the P1 genome region, encoding the structural capsid proteins, when compared to the corresponding regions of human parechoviruses 1 and 2. This has been observed for Ljungan virus as well by us and others [72] and requires more in-depth studies. Only 1 out of 39 ferrets was positive for MpPeV1, suggesting that the ferret parechovirus prevalence may be relatively low (<2.5%). In humans, parechovirus infections are most common in young children and the prevalence in adults may be low as well [73]. It is of note that the parechovirus-positive animal did not show any signs of gastroenteritis. Low virus prevalence was observed for the identified ferret papillomavirus as well. Papillomaviruses are, however, not typically detected in fecal material or associated with gastroenteritis like parechoviruses. Although recently a full-length papillomavirus genome was characterized from fecal material of a deer mouse as well [36]. Not much knowledge exists on papillomatosis in ferrets as a disease, or the virus(es) causing them. The impact of papillomavirus infections on ferrets in general and the specific role of MpPV1 thus has to be further addressed.

Like many other mammals, it seems that ferrets also harbor anelloviruses. The ferret anellovirus MpfTTV1 is most closely related to the recently identified anellovirus from pine marten [25]. Based on phylogenetic analysis, we propose that MpfTTV1 should be placed in the proposed new anellovirus genus, *Xitorquevirus*, in analogy to the classification of torque teno viruses in nine genera named *Alpha-, Beta-, Gamma-, Delta-, Epsilon-, Eta-, Iota-, Theta-*, and *Zetatorquevirus*, and the proposed five genera *Kappa-, Lambda-, Mu-, Nu-,* and *Xitorquevirus* [25], [56], [74]. A closely related virus to MpfTTV1 seemed to be present in one other ferret (Table 1) besides ferret 21, resulting in a virus prevalence of ∼5% in this study. Anelloviruses do not generally seem to be as abundantly present in fecal material as in serum, as evidenced by fecal virome studies in different host species [25], [35], [44], [46], [56], [58], [59], [66].

Ferrets are carnivores that are significantly affected by a number of human pathogens and hence are widely used as a small animal model for viral infections [17]–[21]. In addition, they are capable of spreading viral infections to humans, among which influenza A virus and on rare occasions rabies virus [14]–[16]. The characterization of the fecal virome of ferrets provides epidemiological baseline information about pathogens which allows the swift identification of possible sources of future zoonotic infections and their subsequent control. Especially the seemingly interesting correlation between certain viral infections in ferrets and their presence in human households as pets needs to be further addressed. In addition, this study shows that ferrets can be infected with a wide range of different viruses, some of which may influence the outcome in experimental virus infection studies, thus delivering a strong argument in favor of development of specific pathogen-free ferret colonies.

## Supporting Information

Table S1 **GenBank accession numbers of representative parecho-, papilloma-, and anellovirus strains.**
(DOC)
