# Supplementary material for: Metagenomic Analysis of the Ferret Fecal Viral Flora
Source: PLoS One. 2013 Aug 20;8(8):e71595. doi: 10.1371/journal.pone.0071595 (PMC3748082; doi:10.1371/journal.pone.0071595)
Supplement: Table S1 — GenBank accession numbers of representative parecho-, papilloma-, and anellovirus strains. (DOC) [file pone.0071595.s001.doc]

Table S1. GenBank accession numbers of representative parecho-, papilloma-, and anellovirus strains.

**Virus Abbreviation Genbank accession**

Human parechovirus 1 HPeV1 JX575746

Human parechovirus 2 HPeV2 AF055846

Human parechovirus 3 HPeV3 JX826607

Human parechovirus 4 HPeV4 AM235750

Human parechovirus 5 HPeV5 JX050181

Human parechovirus 6 HPeV6 HQ696577

Human parechovirus 7 HPeV7 EU556224

Human parechovirus 8 HPeV8 EU716175

Ljungan virus NC_003976

Human rhinovirus A 86 HRV-A 86 NC_001617

Human papillomavirus 1 HPV1 V01116

Human papillomavirus 4 HPV4 NC_001457

Human papillomavirus 5 HPV5 M17463

Human papillomavirus 32 HPV32 NC_001586

Human papillomavirus 41 HPV41 NC_001354

Canis familiaris papillomavirus 2 CPV2 NC_006564

Canis familiaris papillomavirus 3 CPV3 NC_008297

Canis familiaris papillomavirus 6 CPV6 NC_013237

Canis familiaris papillomavirus 7 CPV7 FJ492742

Canis familiaris papillomavirus 9 CPV9 JX141478

Canis familiaris papillomavirus 13 CPV13 NC_016974

European elk papillomavirus AaPV1 NC_001524

*Sus scrofa* papillomavirus 1 SsPV1 NC_011280

*Francolinus leucoscepus* papillomavirus 1 FlPV1 NC_013117 *Erinaceus europaeus* papillomavirus 1 EePV1 NC_011765

*Equus caballus* papillomavirus 1 EcPV1 NC_003748

*Equus caballus* papillomavirus 2 EcPV2 NC_012123

*Felis domesticus* papillomavirus 2 FcaPV2 EU796884

*Caretta caretta* papillomavirus 1 CcPV1 NC_011530

Bovine papillomavirus 3 BPV3 NC_004197

Bovine papillomavirus 4 BPV4 X05817

Bovine papillomavirus 5 BPV5 NC_004195

Bovine papillomavirus 6 BPV6 AJ620208

*Fringilla coelebs* papillomavirus FcPV1 NC_004068

*Mastomys natalensis* papillomavirus MnPV1 NC_001605

Cottontail rabbit papillomavirus SfPV1 NC_001541

*Ursus maritimus* papillomavirus 1 UmPV1 NC_010739

*Capra hircus* papillomavirus 1 ChPV1 NC_008032

*Phocoena spinipinnis* papillomavirus PsPV1 NC_003348

Hamster oral papillomavirus MaPV1 E15111

*Rousettus aegyptiacus* papillomavirus 1 RaPV1 NC_008298

*Trichechus manatus* papillomavirus 1 TmPV1 NC_006563

*Erethizon dorsatum* papillomavirus 1 EdPV1 NC_006951

*Psittacus erithacus* timneh papillomavirus PePV1 NC_003973

*Tursiops truncatus* papillomavirus 1 TtPV1 NC_011109

*Odocoileus virginianus* papillomavirus 1 OvPV1 NC_001523

*Rangifer tarandus* papillomavirus 1 RtPV1 AF443292

*Rangifer tarandus* papillomavirus 2 RtPV2 KC810012

*Homo sapiens* torque teno virus 1 HsTTV1 NC_002076

*Homo sapiens* torque mini teno virus 1 HsTTMV1 NC_014097

*Homo sapiens* torque midi teno virus 1 HsTTMDV1 NC_009225

*Aotus trivirgatus* torque teno virus 1 AtTTV 1 NC_014087

*Saguinus oedipus* torque teno virus SoTTV NC_014085

*Sus scrofa* torque teno virus 1 SsTTV1 AY823990

*Sus scrofa* torque teno virus k2 SsTTV k2 NC_014092

*Tupaia belangeri* torque teno virus TbTTV AB057358

*Canis familiaris* torque teno virus CfTTV10 NC_014071

*Felis catus* torque teno virus FcTTV EF538877

*Zalophus californianus* torque teno virus 1 ZcTTV1 NC_012126

*Martes martes* torque teno virus 1 MmTTV1 JN704611
